# Combined Effects of Physical Function and Overall Condition on Functional Independence Measure Gains in Rehabilitation Ward Patients During the Recovery Phase

**DOI:** 10.7759/cureus.98763

**Published:** 2025-12-08

**Authors:** Daisuke Kimura, Hiroki Bizen, Kenta Kunoh, Daiki Nakashima, Teruhumi Iitsuka

**Affiliations:** 1 Department of Rehabilitation, Faculty of Health Sciences, Naragakuen University, Nara, JPN; 2 Department of Occupational Therapy, Faculty of Health Sciences, Kansai University of Health Sciences, Osaka, JPN; 3 Department of Rehabilitation, Yamada Hospital, Gifu, JPN

**Keywords:** anemia, cerebrovascular disease, physical function, recovery, rehabilitation

## Abstract

Background and objective

In the context of Japan’s rapidly aging society, there is an increasing demand for effective rehabilitation strategies to support individuals with diminished physical function. This study aimed to identify the optimal combination of systemic condition and physical function indicators that most strongly influence functional independence measure (FIM) gains, a key outcome index in rehabilitation.

Methods

This retrospective study included 31 inpatients (mean age: 71.5 ± 13.6 years) with cerebrovascular or musculoskeletal diseases in a convalescent rehabilitation ward. Systemic condition was assessed using 14 blood biomarkers (e.g., mean corpuscular hemoglobin concentration (MCHC), albumin (Alb), and total protein (TP)), while physical function was evaluated using grip strength, knee extensor strength-to-weight ratio, the six-minute walk test, and the Berg Balance Scale. Random forest models with five-fold cross-validation were used to identify predictors of FIM gain. Feature importance was assessed using the mean decrease in Gini (MDG) index, and a composite score was calculated to rank variable combinations.

Results

The mean FIM gain during hospitalization was 25.0 ± 17.1 points. The strongest individual predictors among systemic condition indicators were MCHC (MDG = 1.3607), Alb level (1.0777), and TP (0.8868). For physical function, the most important variables were balance ability (2.1481), paralytic-side muscle strength (2.0245), and walking ability (1.9111). Composite score analysis showed that the combination of “balance ability + MCHC” had the highest predictive value (score = 0.6253), followed by “non-paralytic grip strength + MCHC” (0.5754) and “balance ability + Alb” (0.559).

Conclusions

Our findings suggest that combining indicators of physical function, especially balance ability and grip strength, with systemic markers such as MCHC and Alb can enhance the prediction of functional recovery. The use of such combined indices may facilitate more tailored and efficient rehabilitation interventions. Notably, MCHC, a lesser-studied indicator in rehabilitation, showed high predictive value, highlighting the potential role of anemia-related biomarkers in optimizing functional outcomes. Although the sample size was small, this exploratory analysis provides hypothesis-generating insights that may inform future large-scale rehabilitation studies.

## Introduction

In Japan, the rapid progression of an aging population has led to an increase in the number of elderly individuals requiring assistance in daily living due to age-related declines in physical function and chronic illnesses. In this context, the importance of rehabilitation medicine in medical and nursing care settings has grown significantly, with a growing need to accurately assess the physical and mental functions of patients and intervene based on an assessment of their potential for recovery. Improving the ability to predict functional independence measure (FIM) gains has direct clinical relevance, as it supports individualized rehabilitation planning, optimizes resource allocation, and enhances discharge coordination. Identifying combinations of systemic and physical indicators may therefore contribute to more effective and efficient rehabilitation strategies.

In clinical rehabilitation, the FIM is widely used as a comprehensive assessment tool to evaluate the level of independence individuals have in activities of daily living (ADL). The FIM consists of 13 motor items and five cognitive items, and the total score of the 18 items serves as a standard tool for measuring functional independence. The “FIM gain,” which represents the change in FIM scores from admission to discharge, is utilized as an indicator to quantitatively evaluate the effectiveness of rehabilitation and also serves as a common language in discharge support and interdisciplinary collaboration.

Factors influencing FIM gain have traditionally been reported to include age, medical history, and FIM score at admission. However, recent studies have increasingly focused on its association with overall health status [1] and physical function [2]. In particular, several reports have examined its association with blood test indicators reflecting overall health status. For example, patients whose serum albumin (Alb) levels increased during rehabilitation had higher FIM scores at both admission and discharge and also showed larger daily FIM gains. An increase in Alb is an independent predictor of better functional outcomes, suggesting that nutritional status and protein supplementation may have a positive impact on functional recovery [3]. Additionally, patients with hyponatremia have limited improvement in FIM scores for motor and cognitive function after stroke, and it has been noted that sodium concentration negatively impacts FIM gains [4]. Furthermore, studies indicate that elevated alanine aminotransferase (ALT) levels are associated with improved FIM outcomes in elderly patients after hip fractures [5].

Similarly, there are numerous reports on the association between physical function and FIM gains. Improvements in physical elements such as activity level, treatment time, and muscle quality reflect ADL recovery and are consistently associated with increases in FIM scores [1]. Longer daily rehabilitation times are associated with greater improvements in FIM scores, and it has been suggested that the daily duration of occupational or physical therapy may contribute to improvements in functional independence [6]. Additionally, higher FIM scores at admission are predicted to be associated with shorter hospital stays, weekly increases in FIM scores, and a higher likelihood of discharge to home, indicating that initial physical function is a strong predictor of rehabilitation outcomes [7].

Therefore, FIM gain is not merely a change in ADL scores but is closely related to overall physical condition as indicated by blood tests, as well as muscle strength and activity levels, making it an effective indicator for comprehensively assessing a patient’s recovery potential. While many previous studies have analyzed these factors individually, in actual clinical settings, overall physical condition and physical function are believed to interact with each other to influence FIM gain. Thus, comprehensively assessing both factors and clarifying the impact of their combination on FIM gains could provide insights that contribute to the development of more effective and efficient rehabilitation strategies.

The primary objective of this exploratory study was to identify combinations of systemic condition indicators and physical function measures that are most strongly associated with FIM gains, using a composite scoring approach derived from machine learning-based variable importance.

## Materials and methods

We included 31 patients (19 males and 12 females; age: 71.5 ± 13.6 years) admitted to the rehabilitation ward of our hospital. The inclusion criteria were patients with cerebrovascular disease who were admitted to the rehabilitation ward. The exclusion criteria were missing data, transfer to another hospital, and death upon discharge.

For analysis, separate datasets were created for general condition and physical function. General condition indicators included the following 14 blood test items: red blood cell (RBC) count, hematocrit (Ht), platelet (PLT) count, mean corpuscular hemoglobin concentration (MCHC), mean corpuscular volume (MCV), mean corpuscular hemoglobin (MCH), white blood cell (WBC) count, Alb, total protein (TP), ALT, aspartate aminotransferase (AST), CRP, blood urea nitrogen (BUN), and creatinine (Cre).

Physical function indicators included the following six items: grip strength (affected side/non-affected side), knee extensor strength-to-body-weight ratio (affected side/non-affected side), six-minute walk test (6MWT), and Berg Balance Scale (BBS) [8]. The BBS is a widely used balance assessment tool in Japan that does not require special equipment and is simple to administer; hence, it is considered effective for evaluating balance function in daily life.

The outcome variable was defined as the FIM gain (discharge FIM minus admission FIM). Participants were classified into two groups (high and low) based on the median value. For each dataset of general condition and physical function, a classification model was constructed using Random Forest, and important features (variables) for classifying FIM gain were extracted. The mean FIM gain was 26.0 ± 17.1 (range: 3-57) points. Participants were divided into high (≥26 points) and low (<26 points) FIM gain groups based on the median value.

Prior to model construction, data cleaning and preprocessing procedures were conducted. Missing data were handled using predefined exclusion criteria, and therefore, no missing values remained in the analytical dataset. Outliers were assessed through visual inspection of distributions as well as z-scores, and no extreme outliers requiring removal were identified. Because Random Forest is robust to differences in variable scales and does not require normalization or standardization, all variables were entered using their original units without transformation. These preprocessing steps ensured that the data were suitable for machine learning analysis. Given the relatively large SDs observed in several variables, additional outlier screening was performed. Distributions were inspected visually, and z-scores were calculated to detect extreme values. No data points exceeded conventional thresholds (e.g., |z| > 3), and no biologically implausible values were identified. Therefore, all observations were retained for analysis.

The FIM assesses independence on a 7-point scale ranging from full independence to total dependence. Only motor items were analyzed. The FIM is widely used in clinical settings in Japan, and its application for research purposes does not require additional permission. We confirm that no copyrighted materials were reproduced, and only the scoring data routinely collected in clinical practice were analyzed. The FIM was developed by the Uniform Data System for Medical Rehabilitation, a division of UB Foundation Activities Inc., which holds its copyright. Although no certificate of permission is issued in Japan, FIM Version 3 is approved for research use within Japan. The FIM used in this study conforms to Version 3.

Model construction utilized five-fold cross-validation. Participants were randomly divided into five folds, with each fold serving as test data and the remaining data as training data, and the model was repeatedly trained and validated. Random Forest is a machine learning model that improves accuracy by repeatedly performing decision-tree analysis, which represents the necessary items and criteria for FIM gain classification in the form of trees [9,10]. The contribution of each feature to the classification was evaluated using Mean Decrease Gini (MDG), and the average of the five folds was calculated.

Given the exploratory purpose of this study and the small sample size, Random Forest was adopted as a hypothesis-generating approach to efficiently screen a relatively large number of systemic and physical function indicators. Random Forest can capture nonlinear associations and interactions without prior specification of model structure. However, we acknowledge that the use of Random Forest in small datasets may increase the risk of overfitting and instability in variable-importance estimates. Sensitivity analyses comparing Random Forest with linear, logistic, or penalized regression models were considered; however, due to the limited sample size (n = 31) and the large number of predictors relative to the sample, such analyses could not be reliably performed. This limitation was therefore documented explicitly to guide appropriate interpretation.

Furthermore, weight scores were calculated based on the MDG values of the extracted features, and these were substituted into a predetermined evaluation formula to derive a composite score (weighted score). Finally, each item was ranked in order of importance using the composite score. Because no established methodology exists for integrating systemic condition and physical function indicators into a unified composite score, the weighting coefficients (wi, wj) were derived by normalizing the Mean Decrease Gini (MDG) values obtained from the Random Forest models. Thus, the composite score represents an exploratory, non-validated index intended to facilitate hypothesis generation rather than clinical decision-making. The $G_i$ used in the substitution formula represents the average reduction in the Gini coefficient for the overall state, and $w_i$ is the weight for the overall state. $G_j$ represents the average reduction in the Gini coefficient for physical function, and $w_j$ is the weight for physical function. $I_ij$ indicates the importance (overall score). The substitution formula is as follows:

$I_ij=G_i\cdot w_i+G_j\cdot w_j$

Continuous variables were summarized as mean ± SD, and categorical variables as counts with percentages (N (%)). Sex was reported separately for males and females as N (%). Statistical analyses were performed using the Random Forest package in R (version 4.3.0) for machine learning procedures, and composite scores were calculated using Microsoft Excel (Microsoft Corporation, Redmond, WA, USA).

## Results

Descriptive statistics of the participants are presented in Table 1. The mean age of the participants was 71.5 ± 13.6 years, and 61.0% were male. The average values for each parameter were as follows: RBC count (×10⁶/mm³), 427.1 ± 51.0; Ht (%), 39.5 ± 4.3; PLT count (×10⁴/µL), 22.7 ± 7.3; MCHC (g/dL), 32.8 ± 1.0; MCV (fL), 92.8 ± 3.6; MCH (pg), 30.5 ± 1.3; WBC count (/µL), 5990.9 ± 1424.9; Alb (g/dL), 3.8 ± 0.4; TP (g/dL), 6.5 ± 0.6; ALT (U/L), 25.0 ± 27.0; AST (U/L), (value missing in original); CRP (mg/dL), 0.2 ± 0.3; BUN (mg/dL), 15.6 ± 6.1; Cre (mg/dL), 0.8 ± 0.2; affected-side grip strength (kg), 17.1 ± 13.1; non-affected-side grip strength (kg), 24.9 ± 13.1; affected-side knee extension muscle strength-to-body weight ratio (Nm/kg), 29.5 ± 14.3; non-affected-side knee extension muscle strength-to-body weight ratio (Nm/kg), 40.5 ± 17.5; 6MWT (m), 273.9 ± 165.7; and BBS (score), 42.1 ± 15.2.

**Table 1:** Descriptive statistics of the participants Continuous variables are presented as mean ± SD, and categorical variables as N (%). 6MWT, six-minute walk test; Alb, albumin; ALT, alanine aminotransferase; AST, aspartate aminotransferase; BBS, Berg Balance Scale; BUN, blood urea nitrogen; Cre, creatinine; Ht, hematocrit; MCH, mean corpuscular hemoglobin; MCHC, mean corpuscular hemoglobin concentration; MCV, mean corpuscular volume; PLT, platelet; RBC, red blood cell; TP, total protein

Variable	Value
Age (years), mean ± SD	71.5 ± 13.6
Sex, N (%)	-
Male	12 (39%)
Female	19 (61%)
RBC count (×10⁶/mm³)	427.1 ± 51.0
Ht (%)	39.5 ± 4.3
PLT count (×10⁴/µL)	22.7 ± 7.3
MCHC (g/dL)	32.8 ± 1.0
MCV (fL)	92.8 ± 3.6
MCH (pg)	30.5 ± 1.3
WBC count (/µL)	5990.9 ± 1424.9
Alb (g/dL)	3.8 ± 0.4
TP (g/dL)	6.5 ± 0.6
ALT (U/L)	25.0 ± 27.0
AST (U/L)	23.0 ± 12.0
CRP (mg/dL)	0.2 ± 0.3
BUN (mg/dL)	15.6 ± 6.1
Cre (mg/dL)	0.8 ± 0.2
Affected-side grip strength (kg)	17.1 ± 13.1
Non-affected-side grip strength (kg)	24.9 ± 13.1
Affected-side knee extension muscle strength-to-body weight ratio (Nm/kg)	29.5 ± 14.3
Non-affected-side knee extension muscle strength-to-body weight ratio (Nm/kg)	40.5 ± 17.5
6MWT (m)	273.9 ± 165.7
BBS (score)	42.1 ± 15.2

In this study, we calculated the variable importance (MDG) obtained using Random Forest and the weighting score for each item related to general condition and physical function, and then derived a composite score based on these values.

**Figure 1:**
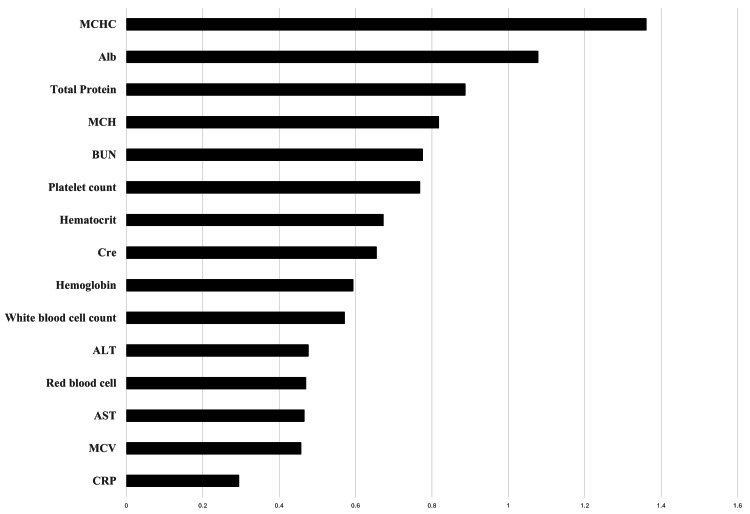
Importance of general condition Alb, albumin; ALT, alanine aminotransferase; AST, aspartate aminotransferase; BUN, blood urea nitrogen; Cre, creatinine; MCH, mean corpuscular hemoglobin; MCHC, mean corpuscular hemoglobin concentration; MCV, mean corpuscular volume

The variable importance of the general condition is shown in Figure 1. Among the general condition items, MCHC (1.3607) had the highest MDG, followed by Alb (1.0777) and TP (0.8868). The corresponding weighting scores were MCHC, 0.1316; Alb, 0.1042; and TP, 0.0857. The overall model accuracy was 0.82 (five-fold cross-validation).

**Figure 2:**
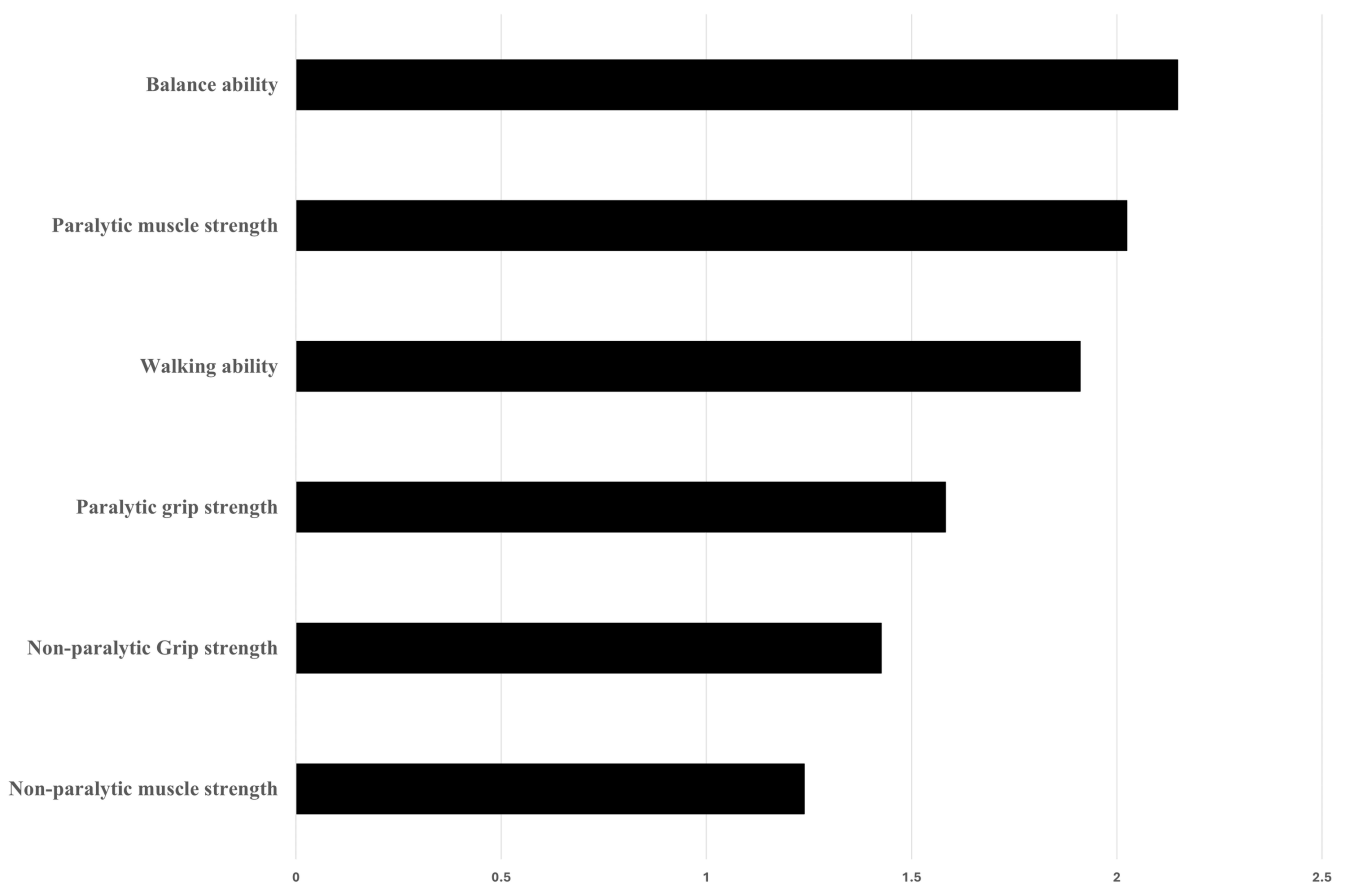
Importance of physical function

The variable importance of physical function is shown in Figure 2. Among the physical function items, balance ability (BBS) (2.1481), paralytic muscle strength (2.0245), and walking ability (6MWT) (1.9111) were ranked in order of importance, with weighting scores of 0.2077, 0.1958, and 0.1848, respectively. The overall model accuracy was 0.79 (five-fold cross-validation).

**Figure 3:**
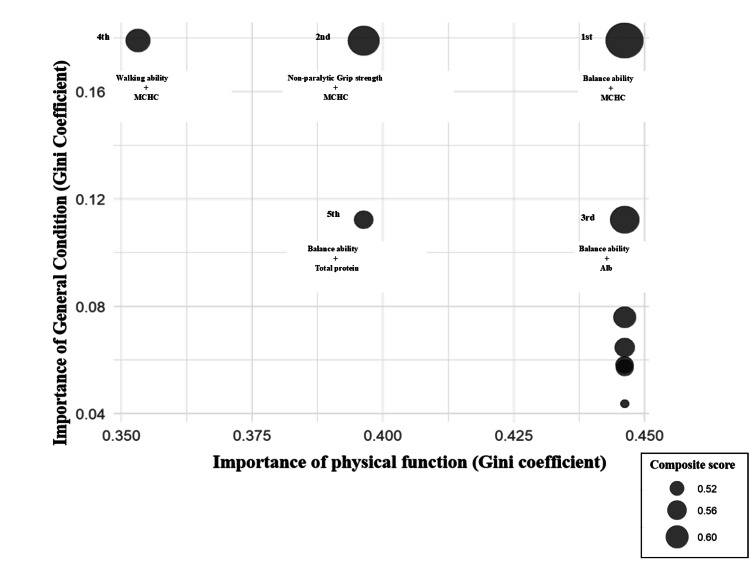
Composite score This bubble plot illustrates the relative contributions of systemic condition and physical function indicators to FIM gain. The x-axis represents the importance (MDG) of physical function variables, and the y-axis represents the importance of systemic condition variables. The size of each bubble corresponds to the composite score derived from the normalized Gini coefficients of both domains. Combinations with larger bubbles indicate higher composite scores, reflecting a stronger joint contribution of physical function and systemic condition to FIM gain. FIM, functional independence measure; MDG, Mean Decrease Gini

Composite scores of major indicators are compared in Figure 3. The combination with the highest composite score was “balance ability + MCHC” (0.6253), followed by “non-paralytic grip strength + MCHC” (0.5754) and “balance ability + Alb” (0.5590). These results suggest combinations of factors highly correlated with FIM gain from both physical function and overall health perspectives. Because of the small sample size and the relatively large number of variables, additional sensitivity analyses using linear, logistic, or penalized regression models could not be performed in a statistically reliable manner. Thus, we were unable to directly compare the robustness of Random Forest with conventional regression-based approaches. This methodological constraint should be considered when interpreting the stability of the identified predictors.

## Discussion

This study explored whether more accurate predictions of FIM gains could be achieved by comprehensively assessing both systemic condition, based on blood test indicators, and physical function. Using Random Forest, we identified the following combinations as effective for predicting FIM gains: balance ability and MCHC, non-paralytic grip strength and MCHC, balance ability and Alb, walking ability and MCHC, and balance ability and TP.

These results suggest that combining specific indicators of overall condition and physical function may be effective in explaining FIM gains. Among the physical function indicators, balance ability, walking ability, and non-paralytic grip strength were extracted. Among these, balance ability was included in three combinations and has been highlighted in previous studies as a physical function strongly associated with ADL [11,12]. Additionally, non-paralytic grip strength is considered an indicator contributing to the maintenance of ADL function in patients with stroke and is associated with a reduced risk of ADL impairment [13,14].

Blood test indicators can be broadly categorized into Alb and TP, which reflect nutritional status, and MCHC, RBC count, and Ht, which are indicators of anemia. In particular, numerous studies have reported that Alb is consistently associated with ADL performance capacity, with higher Alb levels associated with better rehabilitation outcomes and lower levels associated with difficulty in improving ADL [15,16].

Few prior studies have demonstrated a direct association between anemia-related indicators, such as MCHC, and rehabilitation outcomes. However, these indicators are clinically used for diagnosing anemia, and multiple studies have reported that anemia itself is associated with impaired ADL and reduced rehabilitation outcomes [15-18]. Particularly in the elderly and patients with stroke, anemia is known to cause reduced oxygen-carrying capacity, leading to generalized fatigue and decreased exercise tolerance. Patients without anemia are reported to show greater improvements in ADL and higher rehabilitation efficiency.

A distinctive feature of this study is that it explored the potential contribution of anemia-related markers, in addition to conventional nutritional indicators, in predicting FIM gains. This perspective provides useful insights for comprehensively assessing the overall condition of patients and designing individualized rehabilitation interventions.

While the importance of managing nutritional status in rehabilitation is widely recognized, this study goes further by highlighting the signs of anemia and indicating the need for a multifaceted assessment of health status using blood markers. Because anemia may hinder rehabilitation outcomes, incorporating the presence or absence of anemia alongside nutritional status in future intervention designs may contribute to the development of more effective treatment strategies.

This study had several limitations, including a small sample size and its exploratory nature using machine learning. Therefore, the results should be interpreted as hypothesis-generating insights, and future large-scale validation studies are necessary to confirm reproducibility and clinical validity. Given the small sample size (n = 31), the generalizability of the model is limited, and the variable importance values may be unstable due to sampling variability. Thus, the findings should be interpreted with caution, and future research with larger and more diverse cohorts is warranted to validate these preliminary results.

Furthermore, this study has methodological limitations related to the use of Random Forest in a small-sample exploratory context. Although Random Forest is suitable for screening nonlinear patterns, the small sample size increases the possibility of overfitting and decreases the stability of variable importance estimates. Moreover, we considered performing sensitivity analyses using linear, logistic, and penalized regression models to assess the robustness of the findings; however, the number of predictors relative to the small sample size precluded reliable estimation using these methods. Consequently, we were unable to cross-validate the Random Forest-derived predictors through alternative modeling approaches. Future studies with larger cohorts will therefore be necessary to enable such comparative analyses and validate the stability and generalizability of the present findings.

In addition, stroke severity and lesion characteristics, such as NIHSS, mRS, and lesion type or location, were not collected in a standardized manner in this retrospective dataset. Because these factors are well-known determinants of functional recovery, the absence of these variables may have introduced unmeasured confounding. Thus, future studies should incorporate these clinical characteristics to enable more robust and comprehensive modeling.

Moreover, the composite score developed in this study has not been validated, and the weighting method is based solely on normalized MDG values. Therefore, the score should be interpreted as an experimental index designed to explore potential combinations of predictors, rather than as a clinically applicable tool. Future studies are needed to evaluate the reproducibility and external validity of this scoring approach.

Finally, although no extreme outliers were detected using standard criteria, the relatively large variability in some variables, combined with the small sample size, may still influence the stability of feature importance estimates. Therefore, the results should be interpreted with caution, and future studies with larger samples are required to confirm the robustness of these findings.

## Conclusions

In this study, we calculated a composite score to predict FIM gains by comprehensively assessing overall condition, as indicated by blood test indicators, and physical function. We found that the most effective combinations for predicting FIM gains were balance ability and MCHC, non-paralytic grip strength and MCHC, balance ability and Alb, walking ability and MCHC, and balance ability and TP. These findings suggest that combining specific indicators of overall health and physical function may be useful for predicting FIM gains with greater accuracy. Future large-scale, multicenter investigations are warranted to validate these findings.
